# Exploratory Immunophenotyping of Programmed cell death-1 (PD-1) Expression and Regulatory T-Cell (Treg) Profiles in Systemic Lupus Erythematosus and Healthy Controls: A Cohort Study with Illustrative Clinical Cases

**DOI:** 10.7150/ijms.131673

**Published:** 2026-06-17

**Authors:** Po-Jen Hsiao, Jeng-Wei Lu, Yi-Jung Ho, Shan-Wen Lui, Ting-Yu Hsieh, Wun-Long Jheng, Anna Blyzniuk, Feng-Cheng Liu

**Affiliations:** 1 Division of Nephrology, Department of Internal Medicine, Tri-Service General Hospital, National Defense Medical University, Taipei, Taiwan, R.O.C.; 2 Division of Nephrology, Department of Internal Medicine, Taoyuan Armed Forces General Hospital, Taoyuan, Taiwan, R.O.C.; 3 Department of Life Sciences, National Central University, Taoyuan, Taiwan, R.O.C.; 4 Department of Bioscience and Biotechnology, National Taiwan Ocean University, Keelung, Taiwan, R.O.C.; 5 Biotech Research and Innovation Centre, University of Copenhagen, Copenhagen, Denmark.; 6 The Finsen Laboratory, Rigshospitalet/National University Hospital, Faculty of Health and Medical Sciences, University of Copenhagen, Copenhagen, Denmark.; 7 School of Pharmacy, National Defense Medical University, Taipei, Taiwan, R.O.C.; 8 Graduate Institute of Life Sciences, National Defense Medical University, Taipei, Taiwan, R.O.C.; 9 Department of Internal Medicine, Linkou Chang-Gung Memorial Hospital, Taoyuan, Taiwan, R.O.C.; 10 Department of Internal Medicine, Taichung Veterans General Hospital, Taichung, Taiwan, R.O.C.; 11 Cancer Center, Hualien Tzu-Chi Hospital, Hualien, Taiwan, R.O.C.; 12 Department of Engineering Medicine, Shizuoka Center for Molecular Intelligence, Hamamatsu, Shizuoka, Japan.; 13 School of Medicine, O.O. Bogomolets National Medical University, Kyiv, Ukraine.; 14 Rheumatology/Immunology and Allergy, Department of Internal Medicine, Tri-Service General Hospital, National Defense Medical University, Taipei, Taiwan, R.O.C.

## Abstract

**Background:**

Immune checkpoint signaling and regulatory T cells (Tregs) play essential roles in immune homeostasis and immune dysregulation in autoimmune diseases. Programmed cell death-1 (PD-1) signaling and Treg dysfunction have been implicated in systemic lupus erythematosus (SLE); however, temporal variation in immune regulatory profiles across disease states remains incompletely characterized. This study aimed to characterize PD-1 expression and Treg-associated immune profiles in patients with SLE compared with healthy controls while descriptively exploring selected illustrative clinical immune contexts.

**Methods:**

An exploratory longitudinal immunophenotyping study was conducted using multiparameter flow cytometry. The primary exploratory cohort included healthy Taiwanese controls (n = 20) and patients with SLE (n = 10). PD-1 expression across representative T-cell subsets and distributions of Treg-associated immune populations were assessed. Where available, paired longitudinal samples obtained during clinically active and stable disease states were evaluated descriptively to characterize temporal immune variation. Additional illustrative clinical cases representing infection-related and postoperative inflammatory conditions were retained separately for contextual interpretation. Because of the exploratory design and modest sample size, all statistical analyses were considered exploratory and hypothesis-generating.

**Results:**

Compared with healthy controls, patients with SLE demonstrated broader inter-individual variation in PD-1 expression and Treg-associated immune profiles, including differences in activated and memory-associated Treg phenotypes. Paired longitudinal evaluation suggested temporal immune variation between clinically active and stable disease states in selected individuals, although substantial heterogeneity remained evident across patients. Healthy controls also demonstrated measurable baseline variability in immune marker distributions. Selected illustrative clinical cases demonstrated heterogeneous checkpoint-associated and regulatory immune profiles under infection-related and postoperative inflammatory conditions.

**Conclusion:**

This exploratory longitudinal immunophenotyping study describes variation in PD-1 expression and Treg-associated immune profiles across healthy individuals and patients with SLE while providing descriptive contextual observations under selected inflammatory and postoperative clinical conditions. Because of the exploratory design, modest cohort size, clinical heterogeneity, and absence of functional immune validation, all findings should be interpreted cautiously and considered exploratory and hypothesis-generating rather than confirmatory. Larger longitudinal studies incorporating standardized immune monitoring and functional immune assessment are warranted to clarify the biological and clinical relevance of these preliminary observations.

## Introduction

Immune regulation plays a central role in maintaining host defense, immune tolerance, and tissue homeostasis. Among the key mechanisms governing immune balance, the programmed cell death-1 (PD-1) immune checkpoint functions as a critical inhibitory receptor that modulates T-cell activation and contributes to T-cell exhaustion during chronic immune stimulation 1,2. The PD-1/PD-L1 axis has also been recognized as an important mediator of immune regulation and a major therapeutic target in cancer and immune-mediated diseases 3. In parallel, regulatory T cells (Tregs) represent a fundamental component of immune homeostasis. Early studies demonstrated that CD4⁺CD25⁺ T cells are essential for maintaining self-tolerance 4, and subsequent work identified FoxP3 as a master regulator of Treg development and function 5-7. Together, PD-1 signaling and Treg-mediated suppression constitute interconnected regulatory systems that fine-tune immune responses under both physiological and pathological conditions.

Substantial inter-individual variability in immune responses has been well documented. Population-based studies have shown that immune system composition and responsiveness are influenced by genetic background and environmental exposures, resulting in heterogeneity in immune phenotypes 8-10. In addition, immune regulatory pathways are highly dynamic and responsive to inflammatory and physiological conditions. Clinical and experimental studies have demonstrated descriptive and hypothesis-generating insight into immune regulatory variation across autoimmune and inflammatory conditions 11-16. These observations suggest that PD-1 signaling and Treg-associated immune regulation are dynamic and context-dependent rather than fixed immune characteristics 17-20.

Among autoimmune diseases, systemic lupus erythematosus (SLE) is characterized by profound immune dysregulation involving aberrant T-cell activation, defective immune tolerance, and chronic systemic inflammation. Previous studies have suggested that alterations in PD-1 signaling may contribute to immune dysregulation in SLE, while impaired Treg number or function has been implicated in the loss of self-tolerance and disease progression 21-23. Dysregulation of immune checkpoint pathways may reflect both persistent immune activation and compensatory inhibitory responses, highlighting the potential importance of PD-1 and Treg-associated pathways in disease activity and immune variation. However, despite increasing evidence supporting their biological relevance, temporal variation in PD-1 expression and Treg-associated immune profiles across disease states in SLE remains incompletely characterized. Translating these mechanistic insights into clinical practice requires real-world immunophenotyping data that capture the temporal dynamics of PD-1 and Treg-associated immune profiles across disease states in living patients. Despite accumulating experimental and mechanistic evidence, prospective longitudinal immune profiling studies involving clinically well-characterized SLE cohorts remain limited, particularly in East Asian populations. Bridging this translational gap between laboratory immunology and bedside immune monitoring therefore represents an important motivation for the present study.

To address this gap, we conducted an exploratory longitudinal immunophenotyping study using multiparameter flow cytometry to characterize PD-1 expression and regulatory T-cell-associated immune profiles in patients with SLE compared with healthy controls. Where available, paired longitudinal samples obtained during clinically active and stable disease states were evaluated descriptively to explore temporal immune variation. Selected illustrative clinical cases involving infection-related and postoperative inflammatory conditions were retained separately to provide contextual insight into immune heterogeneity under physiological stress. Given the exploratory nature, modest sample size, and clinical heterogeneity of the study population, all findings are intended to provide descriptive and hypothesis-generating insights rather than support causal or population-level inference, thereby informing future larger and longitudinal investigations.

## Methods

### Study Cohort and Recruitment Strategy

This study was designed as an exploratory longitudinal immunophenotyping analysis to characterize PD-1 expression and Treg-associated immune profiles in patients with SLE compared with healthy reference controls, while descriptively evaluating immune variation across selected clinical contexts. Participants were recruited using a convenience sampling strategy from clinical and institutional settings. The primary exploratory cohort comprised healthy Taiwanese controls and patients with SLE.

Healthy controls (n = 20) were included to provide reference distributions for baseline immune variability. Healthy participants were defined by the absence of acute or chronic inflammatory disease, no recent infection or surgery within 4 weeks, and no use of immunomodulatory or immunosuppressive medications. The SLE cohort included 10 patients fulfilling the 2019 European League Against Rheumatism/American College of Rheumatology (EULAR/ACR) classification criteria for SLE. The paired blood samples obtained during clinically active disease and stable follow-up were evaluated descriptively to explore temporal immune variation within individuals. Among patients with paired longitudinal follow-up, clinical disease activity generally decreased during stable follow-up relative to clinically active disease states. SLEDAI-2K (Systemic Lupus Erythematosus Disease Activity Index 2000) is a validated composite disease activity index in which higher scores indicate greater lupus disease activity, whereas BILAG (British Isles Lupus Assessment Group disease activity index) evaluates organ-specific disease activity according to the physician's intention to treat 24. Where available, paired longitudinal blood samples were obtained during clinically active disease and stable follow-up states, with a median sampling interval of 3.5 months (IQR, 2.0-6.0 months). Relevant clinical variables—including comorbidities, treatment exposure, inflammatory markers, disease duration, disease activity, and lupus nephritis status where applicable—were reviewed to provide clinical context for interpretation.

In addition to the primary exploratory cohort, selected individual clinical cases were retained descriptively as illustrative inflammatory and postoperative immune contexts rather than analytical groups. These included a recovery-phase cellulitis case following surgical debridement and a postoperative case following osteoporotic vertebral compression fracture surgery. These illustrative cases were included solely to provide contextual examples of immune variation and were not incorporated into cohort-level statistical analyses. An additional healthy non-Taiwanese participant of Ukrainian (Caucasian) background was evaluated separately in supplementary analyses to illustrate inter-individual immune variability under non-stressed conditions. This participant was not included in the primary exploratory cohort and was not incorporated into formal statistical comparisons or inferential analyses.

### Study Design and Analytical Framework

An exploratory longitudinal immunophenotyping approach was adopted. The primary objective was to characterize variation in PD-1 expression and Treg-associated immune profiles in patients with SLE compared with healthy controls and to descriptively evaluate temporal immune variation where paired follow-up data were available. Multiparameter flow cytometry was used to assess representative T-cell, Treg-associated, and B-cell populations across the exploratory cohort.

Representative immune markers were selected to evaluate immune checkpoint activity, regulatory immune pathways, and adaptive immune variation relevant to autoimmune immune dysregulation. PD-1 and exhaustion-related markers were included to assess checkpoint-associated immune activity and T-cell differentiation states, whereas Treg-associated markers were used to characterize regulatory immune populations involved in immune tolerance. Representative B-cell markers were additionally incorporated to provide broader contextual assessment of adaptive immune variation.

Because of the exploratory design and modest sample size, the study was not intended to establish causal relationships, define disease-specific immune signatures, or support population-level inference. Instead, all findings are intended to provide descriptive and hypothesis-generating insight into immune regulatory variation across autoimmune disease and selected inflammatory or postoperative contexts 25,26.

Immune marker distributions were interpreted primarily using individual-level visualization approaches, including dot plots, paired longitudinal displays, and integrated heatmap analyses, to preserve inter-individual variability and avoid overinterpretation of summary immune profiles. Selected illustrative clinical cases and supplementary descriptive observations were evaluated separately from the primary exploratory cohort and were not incorporated into inferential cohort-level analyses.

### Flow Cytometry-Based Immunophenotyping

Peripheral venous blood samples were collected in EDTA-containing tubes and processed within 2-4 hours after collection. Peripheral blood mononuclear cells (PBMCs) were isolated using Ficoll-Paque PLUS (GE Healthcare, Uppsala, Sweden), washed, and resuspended in staining buffer for multiparameter flow cytometric analysis.

The gating strategy followed a sequential workflow. Debris and doublets were excluded based on forward and side scatter characteristics, followed by exclusion of non-viable cells using a fixable viability dye. CD3⁺ T cells were identified and subdivided into CD4⁺ and CD8⁺ populations. Regulatory T cells were defined as CD4⁺CD25^highCD127^low/-FoxP3⁺ cells. Treg subsets, including naïve, activated, memory, resting, CD39⁺, Helios⁺, and Tr1-associated populations, were further characterized according to previously established phenotypic definitions and gating strategies 27-31. B-cell subsets were identified within CD19⁺ cells and classified into naïve, switched memory, transitional, plasmablast, and regulatory B-cell populations based on CD27 and IgD expression 32. PD-1, KLRG1, Fas, and Tim-3 expression were evaluated across representative T-cell subsets.

Cells were stained with fluorochrome-conjugated monoclonal antibodies targeting representative T-cell, Treg, and B-cell-associated immune markers, including CD3, CD4, CD8, CD25, CD127, PD-1, KLRG1, Fas, Tim-3, CD19, CD27, and IgD. Intracellular FoxP3 staining was performed using a transcription factor staining buffer set according to the manufacturer's instructions. Detailed antibody information and fluorochrome combinations are provided in Supplementary Table S1.

Data acquisition was performed using multicolor flow cytometers (BD FACSCanto II or BD LSRFortessa; BD Biosciences, San Jose, CA, USA), and analyses were conducted using FlowJo software (Tree Star Inc., Ashland, OR, USA). Instrument settings were standardized across samples. Compensation was performed using single-stained controls, and fluorescence-minus-one (FMO) controls were applied for selected markers, including PD-1 and Treg-associated immune markers.

Additional immune markers associated with T-cell activation, differentiation, exhaustion, and senescence—including KLRG1, Fas, and Tim-3—were evaluated according to prior studies of immune checkpoint signaling and T-cell dysfunction 33-35. Because of the exploratory design and modest cohort size, all immunophenotypic findings were interpreted descriptively and considered hypothesis-generating rather than confirmatory.

### Data Interpretation and Visualization

Immune marker distributions were summarized primarily as proportions and visualized using individual-level dot plots, paired longitudinal plots, and integrated heatmaps. Paired longitudinal visualization was used to descriptively illustrate temporal immune variation between clinically active disease and stable follow-up states in patients with SLE where paired samples were available. Individual-level visualization approaches were prioritized to preserve inter-individual variability and avoid overinterpretation of summarized immune distributions within the modest exploratory cohort.

For heatmap visualization, immune marker data were normalized using row-wise z-score transformation across participants included in the primary exploratory cohort, including healthy controls and patients with SLE. Heatmaps were generated solely for descriptive visualization of immune heterogeneity across representative PD-1-associated, Treg-associated, and B-cell immune markers. Because normalization was performed across the full cohort, values represent relative positioning within the study population rather than within-group variation. Given the exploratory design, modest sample size, and clinical heterogeneity of the cohort, heatmap analyses were intended for descriptive pattern recognition only and should not be interpreted as evidence of disease-specific, mechanistic, statistical, or inferential immune differences.

Selected illustrative clinical cases and supplementary descriptive observations were visualized separately from the primary exploratory cohort and interpreted descriptively to provide contextual examples of immune variation under inflammatory and non-inflammatory conditions. These supplementary observations were not incorporated into primary cohort-level interpretation.

### Statistical Analysis

Because of the exploratory nature of the study and modest sample size, statistical analyses were performed using non-parametric methods. Continuous variables were summarized primarily as median with interquartile range (IQR). Mean ± standard deviation (SD) values were additionally provided for selected descriptive visualization where appropriate.

Exploratory comparisons between healthy controls and patients with SLE were performed using the Mann-Whitney U test. For participants with paired longitudinal follow-up data, within-individual comparisons between clinically active disease and stable follow-up states were evaluated using the Wilcoxon signed-rank test. Where applicable, descriptive associations between representative immune markers and clinical disease activity variables were explored using Spearman's rank correlation coefficient.

Given the exploratory design, modest cohort size, clinical heterogeneity, and absence of functional immune validation, all statistical findings should be interpreted cautiously and considered hypothesis-generating rather than confirmatory. No formal power calculation or adjustment for multiple comparisons was performed.

## Results

### Study Cohort and Clinical Characteristics

**Figure 1:**
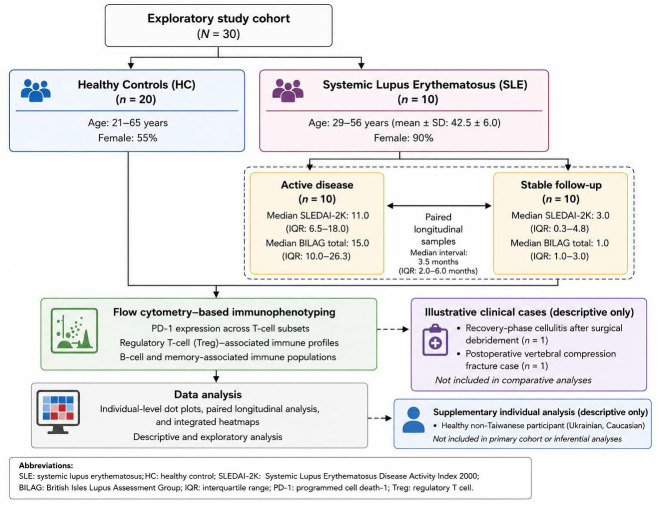
** Study cohort and analytical framework of the exploratory longitudinal immunophenotyping study.** Overview of participant recruitment, cohort composition, longitudinal sampling strategy, and analytical workflow used to characterize immune regulatory profiles in patients with systemic lupus erythematosus (SLE) and healthy controls (HC). The primary exploratory cohort comprised healthy controls (HC, n = 20) and patients with SLE (n = 10), with paired longitudinal follow-up samples obtained during clinically active disease and stable follow-up where available. Clinical variables—including demographics, treatment exposure, inflammatory markers, disease duration, disease activity, lupus nephritis status, and comorbidities—were reviewed for contextual interpretation. Two selected individual clinical cases (n = 2), representing post-infectious recovery and postoperative inflammatory conditions, were retained separately for descriptive contextual visualization and were not included in cohort-level statistical comparisons. Peripheral blood mononuclear cells (PBMCs) were analyzed using multiparameter flow cytometry to assess programmed cell death-1 (PD-1) expression across representative T-cell subsets and regulatory T-cell (Treg)-associated immune profiles. Analytical approaches included descriptive immune profiling, exploratory non-parametric analyses, paired longitudinal visualization, and integrated heatmap analyses to preserve inter-individual immune variability. Findings are intended to provide descriptive and hypothesis-generating insights rather than causal or population-level inference. Abbreviations: HC, healthy controls; SLE, systemic lupus erythematosus; PBMC, peripheral blood mononuclear cell; PD-1, programmed cell death-1; Treg, regulatory T cell; EULAR/ACR, European League Against Rheumatism/American College of Rheumatology; SLEDAI, Systemic Lupus Erythematosus Disease Activity Index.

A total of 30 participants were included in the primary exploratory cohort, comprising healthy controls (n = 20) and patients with SLE (n = 10) (Figure 1). The SLE cohort consisted predominantly of female participants (90%) with a mean age of 42.5 ± 6.0 years and a median disease duration of 4.2 years (IQR, 2.1-10.6). Where available, paired longitudinal blood samples were obtained during clinically active disease and stable follow-up states, with a median sampling interval of 3.5 months (IQR, 2.0-6.0 months). Among patients with paired longitudinal follow-up, clinical disease activity decreased during stable follow-up compared with clinically active disease states, as reflected by reductions in both SLEDAI-2K and BILAG scores. Detailed demographic characteristics, disease manifestations, treatment exposure, and longitudinal disease activity data are summarized in Table 1.

Comprehensive flow cytometry-based immunophenotyping focused on PD-1 expression across representative T-cell subsets and the distribution of Treg-associated immune populations. Immune marker distributions were visualized primarily using individual-level dot plots, paired longitudinal displays, and integrated heatmaps because of the exploratory design and modest cohort size.

### Baseline Immune Variability in Healthy Controls

**Figure 2:**
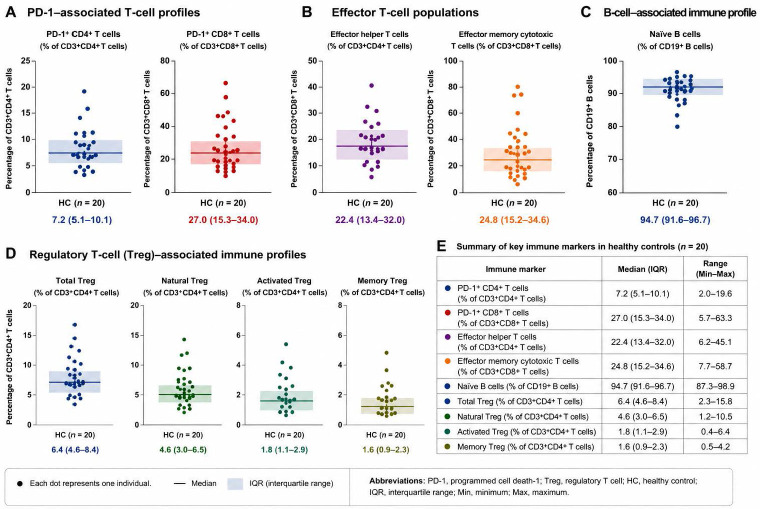
** Baseline immune reference distributions in healthy controls.** Individual-level distributions of key immune markers in healthy controls (HC, n = 20) were evaluated using flow cytometry-based immunophenotyping. Each dot represents one individual participant. Horizontal lines indicate the median, and shaded areas represent the interquartile range (IQR). (A) PD-1-associated T-cell profiles, including PD-1⁺ CD4⁺ T cells and PD-1⁺ CD8⁺ T cells. (B) Effector T-cell populations, including effector helper T cells and effector memory cytotoxic T cells. (C) B-cell-associated immune profile showing naïve B-cell distribution. (D) Regulatory T-cell (Treg)-associated immune profiles, including total Treg, natural Treg, activated Treg, and memory Treg populations. (E) Summary table showing median values, interquartile ranges, and overall ranges for the evaluated immune markers in healthy controls. These analyses were performed to establish reference distributions and illustrate baseline inter-individual immune variability under non-inflammatory conditions. Abbreviations: PD-1, programmed cell death-1; Treg, regulatory T cell; HC, healthy control; IQR, interquartile range.

Among healthy controls, PD-1 expression and Treg-associated immune profiles demonstrated measurable inter-individual variability across multiple immune markers (Figure 2). Effector helper and effector memory cytotoxic T-cell populations showed moderate variation across participants, whereas naïve B-cell populations remained relatively preserved in most individuals. Regulatory T-cell subsets also demonstrated variability, particularly among activated and memory-associated Treg populations, while overall checkpoint-associated and regulatory immune profiles remained relatively stable within healthy reference distributions. These observations provided contextual baseline immune variability for exploratory comparison with patients with SLE.

### PD-1 Expression in SLE and Longitudinal Immune Variation

**Figure 3:**
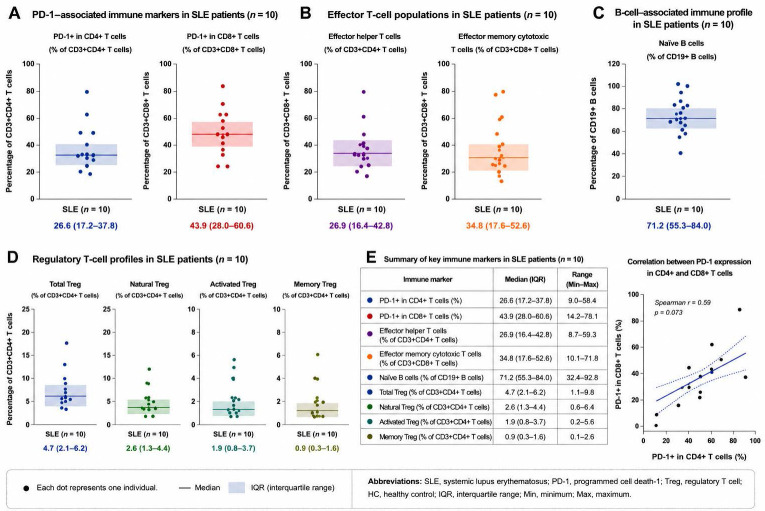
** Immune profiling of patients with systemic lupus erythematosus.** Individual-level distributions of key immune markers in patients with systemic lupus erythematosus (SLE, n = 10) were evaluated using flow cytometry-based immunophenotyping. Each dot represents one individual participant. Horizontal lines indicate the median, and shaded areas represent the interquartile range (IQR). (A) PD-1-associated immune markers, including PD-1⁺ CD4⁺ T cells and PD-1⁺ CD8⁺ T cells. (B) Effector T-cell populations, including effector helper T cells and effector memory cytotoxic T cells. (C) B-cell-associated immune profile showing naïve B-cell distribution. (D) Regulatory T-cell (Treg)-associated immune profiles, including total Treg, natural Treg, activated Treg, and memory Treg populations. (E) Summary table showing median values, interquartile ranges, and overall ranges for the evaluated immune markers in patients with SLE. The correlation plot demonstrates the relationship between PD-1 expression in CD4⁺ and CD8⁺ T cells using Spearman correlation analysis. These analyses were performed to characterize inter-individual immune heterogeneity and exploratory immune checkpoint-associated alterations in patients with SLE. Abbreviations: SLE, systemic lupus erythematosus; PD-1, programmed cell death-1; Treg, regulatory T cell; HC, healthy control; IQR, interquartile range; Min, minimum; Max, maximum.

Patients with SLE demonstrated broader heterogeneity across PD-1-associated immune markers compared with healthy controls. Naïve B-cell distributions also appeared lower and more heterogeneous in several patients with SLE compared with healthy controls (Figure 3). Relative increases in PD-1-associated immune phenotypes were observed in selected patients during clinically active disease states, particularly within effector helper and cytotoxic T-cell populations. Where paired longitudinal samples were available, temporal variation in immune checkpoint-associated profiles were observed between active disease and stable follow-up. In several individuals, reductions in selected PD-1-associated immune markers coincided with lower SLEDAI-2K and BILAG disease activity scores during stable follow-up, although substantial inter-individual variability remained evident.

### Regulatory T-Cell Profiles in SLE

**Figure 4:**
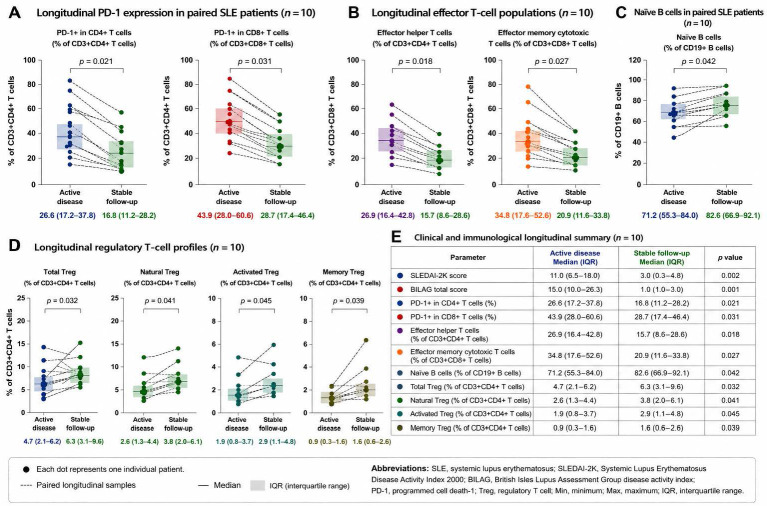
** Longitudinal immune profiling during active disease and stable follow-up in paired systemic lupus erythematosus samples.** Paired longitudinal immune profiling was performed in patients with systemic lupus erythematosus (SLE, n = 10) using flow cytometry-based immunophenotyping. Individual paired blood samples obtained during clinically active disease and stable follow-up were analyzed descriptively to evaluate temporal immune variation within individuals. Each dot represents one individual patient, and dashed lines indicate paired longitudinal samples. Horizontal lines indicate the median, and shaded areas represent the interquartile range (IQR). P values were calculated using the Wilcoxon signed-rank test. (A) Longitudinal PD-1-associated immune markers, including PD-1⁺ CD4⁺ T cells and PD-1⁺ CD8⁺ T cells. (B) Longitudinal effector T-cell populations, including effector helper T cells and effector memory cytotoxic T cells. (C) Longitudinal naïve B-cell profiles. (D) Longitudinal regulatory T-cell (Treg)-associated immune profiles, including total Treg, natural Treg, activated Treg, and memory Treg populations. (E) Clinical and immunological longitudinal summary comparing clinically active disease and stable follow-up states, including SLEDAI-2K scores, BILAG total scores, and evaluated immune markers. Overall, clinically stable follow-up samples demonstrated reduced PD-1-associated and effector T-cell immune activation, accompanied by relative recovery of naïve B-cell and Treg-associated immune profiles. Abbreviations: SLE, systemic lupus erythematosus; SLEDAI-2K, Systemic Lupus Erythematosus Disease Activity Index 2000; BILAG, British Isles Lupus Assessment Group disease activity index; PD-1, programmed cell death-1; Treg, regulatory T cell; IQR, interquartile range; Min, minimum; Max, maximum.

Substantial heterogeneity in Treg-associated immune profiles was observed across healthy controls and patients with SLE (Figure 4). Compared with healthy participants, several patients with SLE demonstrated relatively enriched activated and memory-associated Treg phenotypes during clinically active disease states. Paired longitudinal visualization further suggested dynamic variation in Treg-associated immune profiles between active disease and stable follow-up. In several patients, total Treg, natural Treg, activated Treg, and memory Treg populations demonstrated relative increases during stable follow-up in parallel with lower clinical disease activity. Nevertheless, considerable inter-individual variability remained evident, and heterogeneous longitudinal patterns were observed across individual patients.

### Integrated Analysis of PD-1 and Treg Profiles

**Figure 5:**
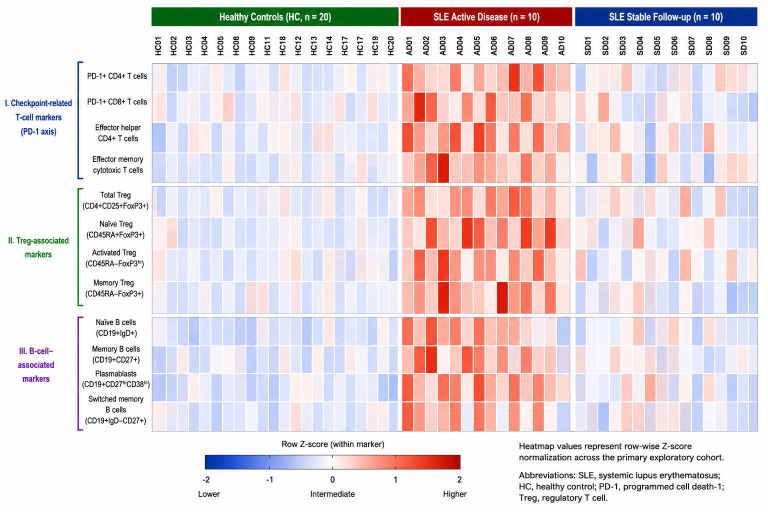
** Integrated heatmap visualization of immune marker distributions across healthy controls and systemic lupus erythematosus disease states.** Integrated heatmap visualization of flow cytometry-based immune profiling across healthy controls (HC, n = 20), patients with active systemic lupus erythematosus (SLE active disease, n = 10), and paired clinically stable follow-up samples (SLE stable follow-up, n = 10). Samples were displayed according to predefined clinical grouping rather than unsupervised hierarchical clustering. Immune markers were functionally grouped into three major categories: (I) checkpoint-related T-cell markers (PD-1 axis), (II) regulatory T-cell (Treg)-associated markers, and (III) B-cell-associated markers. Heatmap values represent row-wise Z-score normalization within each immune marker across the primary exploratory cohort. Red coloration indicates relatively higher expression or abundance, whereas blue coloration indicates relatively lower expression relative to the cohort-wide distribution for each marker. Active SLE samples demonstrated increased PD-1-associated and effector immune activation patterns, while clinically stable follow-up samples showed partial normalization toward healthy control immune distributions. The heatmap illustrates inter-individual immune heterogeneity and longitudinal immune variation across disease activity states in this exploratory immunophenotyping cohort. Abbreviations: SLE, systemic lupus erythematosus; PD-1, programmed cell death-1; Treg, regulatory T cell.

An integrated heatmap incorporating checkpoint-related T-cell markers, Treg-associated immune markers, and B-cell-associated immune populations was generated to visualize overall immune variation across healthy controls and patients with SLE (Figure 5). Healthy controls demonstrated relatively clustered immune distributions despite measurable inter-individual variability, whereas patients with SLE exhibited broader heterogeneity across checkpoint-related, regulatory, and B-cell-associated immune markers. In several patients with SLE, relatively higher positioning of PD-1-associated and Treg-associated immune markers, together with variation in selected B-cell-associated immune populations, was observed during clinically active disease states compared with stable follow-up. Longitudinal visualization further suggested partial normalization of selected immune profiles during stable follow-up, although substantial inter-individual variability remained evident and the magnitude of change varied across patients. Because heatmap visualization was based on row-wise normalized relative positioning rather than inferential statistical analysis, all observations are intended solely for descriptive pattern recognition and hypothesis generation.

### Illustrative Immune Profiles in Selected Clinical Cases

In addition to the primary exploratory cohort, two representative clinical cases were descriptively retained to illustrate contextual immune variation under inflammatory and postoperative conditions. The infection-related case involved a 47-year-old male evaluated during recovery following surgical debridement for post-traumatic cellulitis. This case demonstrated relatively higher positioning of PD-1-associated immune markers across activated helper and cytotoxic T-cell populations compared with healthy reference distributions. Relatively increased activated Treg-associated and CD39⁺ regulatory immune populations were also observed during the recovery phase of localized inflammation (Table 2). In contrast, the postoperative case involved an 88-year-old female evaluated following vertebral compression fracture surgery and demonstrated broader heterogeneity across checkpoint-associated and regulatory immune markers. Compared with the infection-related case, relative positioning of PD-1-associated immune markers appeared less prominent, whereas mixed distributions of activated and memory-associated Treg populations remained evident (Table 2).

An additional healthy participant of Ukrainian background was evaluated separately in supplementary analyses to provide a descriptive illustration of inter-individual immune variability. Compared with the healthy Taiwanese reference cohort, this participant demonstrated lower PD-1⁺ Effector Helper T-cell proportions and higher Memory Treg proportions (Supplementary Figure 1). Serial observations obtained during asymptomatic and mild upper respiratory symptom states further demonstrated temporal fluctuation in these immune markers. Because this supplementary observation was based on a single individual and included solely for descriptive visualization, the findings were not incorporated into formal statistical comparisons, inferential analyses, or cohort-level analyses.

## Discussion

### Baseline Variation in Immune Regulatory Profiles

Healthy controls demonstrated measurable inter-individual variability in PD-1 expression and Treg profiles despite the absence of overt inflammatory illness. Moderate variability was observed across effector T-cell populations and activated or memory Treg subsets, suggesting that baseline immune regulatory states may differ substantially between individuals. These findings are consistent with previous studies showing that immune system composition and responsiveness vary according to genetic background, environmental exposures, antigen experience, microbiome composition, and lifestyle-related factors 8-10,25,26. Accordingly, variation in immune checkpoint signaling and Treg profiles may reflect host-specific immune regulatory characteristics rather than pathological immune dysregulation alone. Previous studies have also demonstrated that immune aging, immunosenescence, T-cell exhaustion, and population-level genetic adaptation can influence immune regulatory pathways and contribute to heterogeneity in immune phenotypes 35-41.

Because of the exploratory design and modest sample size, the determinants underlying immune heterogeneity could not be systematically evaluated. Therefore, these observations should be interpreted as descriptive rather than mechanistic or population-level findings.

### Clinical Implications of Immune Regulatory Variation in SLE

Compared with healthy controls, patients with SLE demonstrated broader heterogeneity across checkpoint-related and regulatory immune markers, including variation in PD-1 expression and activated or memory Treg populations. Paired longitudinal observations further suggested that checkpoint-related and Treg profiles may vary dynamically across clinically active and stable disease states, although substantial inter-individual variability remained evident.

In several individuals, relative reductions in selected PD-1-related markers and activated Treg populations were observed during stable follow-up together with lower SLEDAI-2K and BILAG disease activity scores. However, persistent elevations and heterogeneous longitudinal patterns remained evident in some patients, highlighting the individualized and context-dependent nature of immune regulation in SLE.

These observations are broadly consistent with previous studies implicating T-cell dysregulation, regulatory T-cell imbalance, immune checkpoint pathways, and age-associated immune remodeling in autoimmune and immune-mediated diseases 21-23,38-41. However, because of the exploratory design, modest cohort size, and absence of functional immune validation, the clinical implications of these findings remain preliminary and require confirmation in larger longitudinal cohorts.

### Contextual Immune Variation in Illustrative Clinical Cases

Selected illustrative clinical cases involving post-infectious recovery and postoperative inflammatory conditions were included descriptively to provide contextual insight into immune heterogeneity under clinically relevant settings. Relative variation in PD-1-related markers and Treg profiles was observed compared with healthy reference distributions. The infection-related recovery case demonstrated relatively increased activated and memory Treg populations together with enriched PD-1-related markers across helper and cytotoxic T-cell subsets during recovery following localized cellulitis. In contrast, the postoperative case demonstrated broader heterogeneity across checkpoint-related and regulatory immune markers with mixed activated and memory Treg distributions.

An additional participant of Ukrainian background, evaluated separately in supplementary analyses, further illustrated measurable inter-individual immune variability under different physiological and mild inflammatory conditions. Relative variation in PD-1⁺ Effector Helper T-cell proportions and Memory Treg proportions was observed compared with the healthy Taiwanese reference cohort. Serial observations obtained during asymptomatic and mild upper respiratory symptom states further demonstrated temporal fluctuation in these immune markers. Because this supplementary observation was based on a single individual and was included solely for descriptive visualization, the findings should be interpreted cautiously as contextual observations rather than evidence of ethnicity-associated, population-level, or disease-specific immune differences.

Because these observations were derived from individual cases with heterogeneous clinical conditions and without standardized longitudinal validation, they should be interpreted cautiously as descriptive contextual findings rather than evidence of condition-specific or population-level immune responses.

### Strengths and Limitations

This study has several strengths. First, the study adopted an exploratory longitudinal immunophenotyping framework to characterize PD-1 expression and Treg-associated immune profiles in patients with SLE relative to healthy controls, providing contextual insight into baseline immune variability. Second, the use of multiparameter flow cytometry enabled detailed characterization of PD-1 expression and multiple Treg-associated immune populations across representative immune regulatory compartments. Third, paired longitudinal immune profiling available in selected patients with SLE provided preliminary insight into temporal immune variation across clinically active and stable disease states. Fourth, individual-level visualization approaches—including paired longitudinal plots and integrated heatmaps—helped preserve inter-individual immune heterogeneity and facilitated descriptive interpretation within a modest exploratory cohort. Finally, the inclusion of illustrative inflammatory, postoperative, and supplementary contextual observations provided additional perspective on immune variability across distinct clinical settings and mild inflammatory conditions.

Several limitations should also be acknowledged. First, despite inclusion of a primary exploratory cohort, the overall sample size remained modest, limiting statistical power and generalizability. Second, the SLE cohort was clinically heterogeneous, and important confounding factors—including disease activity, medication exposure, comorbidities, inflammatory status, disease duration, and potential age and gender imbalance relative to healthy controls—may have influenced immune phenotypes and were not uniformly controlled. Third, although longitudinal follow-up data were available in selected patients, sampling intervals and treatment conditions were not fully standardized. Fourth, functional assays evaluating T-cell exhaustion, cytokine activity, suppressive immune function, or mechanistic signaling pathways were not performed; therefore, the observed immunophenotypic findings should not be interpreted as direct evidence of functional immune dysregulation. Fifth, statistical analyses were exploratory in nature, and no adjustment for multiple comparisons was performed because of the hypothesis-generating design and modest sample size of the study. Finally, the illustrative clinical cases and supplementary descriptive observations—including the additional participant of Ukrainian background—were based on individual-level observations and were included solely for contextual interpretation rather than inferential comparison.

## Conclusion

This exploratory longitudinal immunophenotyping study describes inter-individual variation in PD-1 expression and Treg-associated immune profiles across healthy individuals and patients with SLE. Longitudinal observations further suggested dynamic immune variation between clinically active disease and stable follow-up states, highlighting the individualized and context-dependent nature of immune regulation in SLE.

Because of the exploratory design, modest cohort size, clinical heterogeneity, and absence of functional immune validation, these findings should be interpreted cautiously and considered hypothesis-generating rather than confirmatory. Nevertheless, the present study provides preliminary descriptive insight into immune regulatory variation in SLE and supports further investigation in larger, well-characterized longitudinal cohorts with standardized immune monitoring and functional immune assessment.

## Supplementary Material

Supplementary figure and table.

## Figures and Tables

**Table 1:** Baseline demographic and clinical characteristics of patients with systemic lupus erythematosus

Variable	SLE cohort (n = 10)
Sex, female, n (%)	9 (90)
Age, years	42.5 ± 6.0
Disease duration, years	4.2 (2.1-10.6)
Lupus nephritis, n (%)	3 (30)
Pulmonary arterial hypertension, n (%)	2 (20)
Sjögren syndrome/interstitial lung disease, n (%)	1 (10)
Cerebrovascular involvement with suspected antiphospholipid syndrome, n (%)	1 (10)
Opportunistic infection history, n (%)	1 (10)
Corticosteroid treatment, n (%)	10 (100)
Immunosuppressive treatment, n (%)	8 (80)
Hydroxychloroquine treatment, n (%)	7 (70)
SLEDAI-2K score during active disease	11.0 (6.5-18.0)
SLEDAI-2K score during stable follow-up	3.0 (0.3-4.8)
BILAG total score during active disease	15.0 (10.0-26.3)
BILAG total score during stable follow-up	1.0 (1.0-3.0)

Abbreviations: SLE, systemic lupus erythematosus; SLEDAI-2K, Systemic Lupus Erythematosus Disease Activity Index 2000; BILAG, British Isles Lupus Assessment Group; APS, antiphospholipid syndrome. Data presentation: Data are presented as mean ± standard deviation (SD), median (interquartile range [IQR]), or number (%), as appropriate. Immunosuppressive agents included mycophenolate mofetil, azathioprine, tacrolimus, and cyclophosphamide according to individual clinical indications.

**Table 2:** Clinical characteristics (A) and immunophenotypic profiles (B) of two representative clinical stress conditions.

(A) Clinical Characteristics			
Variable	Cellulitis (47-year-old male)	Elderly Postoperative (88-year-old female)	
Demographics	Previously healthy adult	Elderly individual with multiple comorbidities	
Primary condition	Post-traumatic cellulitis (right foot)	Osteoporotic vertebral fracture, postoperative state	
Clinical context	Acute infection with surgical intervention	Chronic inflammation, aging, recurrent infection	
Comorbidities	None documented	Paraplegia, pressure ulcers, UTI, osteoporosis, GERD, depression	
Type of stress	Acute inflammation + debridement	Chronic inflammation + surgery + aging	
Clinical course	Infection → CRP elevation → debridement → antibiotics → recovery	Surgery → immobilization → pressure ulcers → recurrent infection	
Treatment exposure	Debridement + antibiotics (ampicillin/sulbactam)	Multiple surgeries + antibiotics + long-term care	
CRP (mg/dL)	4.5	7.8	
WBC (×10³/μL)	10.54	9.65	
Infection status	Localized infection	Chronic/recurrent infection	
Timing of sampling	Post-infection recovery phase	Postoperative with ongoing inflammation	
(B) Immunophenotypic Profiles			
Immune Parameter			Descriptive Interpretation
PD-1 expression	Increased relative to baseline	Higher relative levels observed	Consistent with checkpoint activation
Naïve T cells	Slightly lower relative levels	Lower relative levels observed	Compatible with age-related changes
Effector T cells	Higher relative levels	Lower relative levels	Distinct response patterns observed
Memory Tregs	Higher relative levels	Higher relative levels	Regulatory changes observed
Activated Tregs	Slightly higher relative levels	Higher relative levels	Sustained regulatory activity
Natural Tregs	Similar to or slightly higher	Higher relative levels	Compatible with aging-related remodeling
CD39⁺ Tregs	Higher relative levels	Higher relative levels	Suggestive of suppressive phenotype
Regulatory B cells	Slightly higher relative levels	Higher relative levels	Possible compensatory regulation
Plasmablasts	Similar to or slightly higher	Higher relative levels	Consistent with ongoing stimulation
Tr1 cells	Higher relative levels	Higher relative levels	IL-10-associated regulatory response

Abbreviations: CRP, C-reactive protein; WBC, white blood cell count; UTI, urinary tract infection; GERD, gastroesophageal reflux disease; Tregs, regulatory T cells. Reference ranges: CRP, <0.5 mg/dL; WBC count, 4.0-10.0 × 10³/μL All data are presented descriptively without formal statistical analysis due to the small sample size. Immunophenotypic findings represent relative differences compared with baseline observations and should be interpreted as exploratory and hypothesis-generating. Clinical variables were obtained from available medical records at the time of sampling.

## Data Availability

The datasets used and/or analyzed during the current study are available from the corresponding author on reasonable request.

## Abbreviations

HC: healthy controls
PD-1: programmed cell death-1
Treg: regulatory T cell
SD: standard deviation
