# Supplementary material for: Exploratory Immunophenotyping of Programmed cell death-1 (PD-1) Expression and Regulatory T-Cell (Treg) Profiles in Systemic Lupus Erythematosus and Healthy Controls: A Cohort Study with Illustrative Clinical Cases
Source: Int J Med Sci. 2026 Jun 17;23(8):2511–23. doi: 10.7150/ijms.131673 (PMC13411475; doi:10.7150/ijms.131673)
Supplement: Supplementary file 1 — Supplementary figure and table. [file ijmsv23p2511s1.pdf]

Supplementary Table S1. Antibodies and immune markers used for flow cytometric immunophenotyping. Representative T-cell, regulatory T-cell (Treg), and B-cell markers were selected based on established immunophenotyping strategies and published definitions of human Treg subsets.

Marker	Fluorochrome	Clone	Manufacturer	Catalog No.
CD3	APC-H7	SK7	BD Biosciences	560176
CD4	FITC	RPA-T4	BD Biosciences	555346
CD8	PE-Cy7	RPA-T8	BD Biosciences	557746
CD25	APC	M-A251	BD Biosciences	555434
CD127	PE	HIL-7R-M21	BD Biosciences	557938
PD-1	PE	EH12.2H7	BioLegend	329906
KLRG1	FITC	SA231A2	BioLegend	367706
Fas (CD95)	APC	DX2	BD Biosciences	555674
Tim-3	PE	F38-2E2	BioLegend	345006
CD19	APC-H7	SJ25C1	BD Biosciences	560295
CD27	PE	M-T271	BD Biosciences	555441
IgD	FITC	IA6-2	BD Biosciences	555778
CD24	PerCP-Cy5.5	ML5	BD Biosciences	561646
CD38	APC	HIT2	BD Biosciences	555462
FoxP3	Alexa Fluor 488	PCH101	eBioscience (Thermo Fisher)	53-4776-42
Helios	PE	22F6	BioLegend	137204
CD39	APC	A1	BioLegend	328206
Viability Dye	Fixable Viability Dye	—	BioLegend	423101

Abbreviations: APC, allophycocyanin; FITC, fluorescein isothiocyanate. Compensation was performed using single-stained controls. Fluorescence-minus-one (FMO) controls were applied for key markers. Viability dye was used to exclude dead cells.

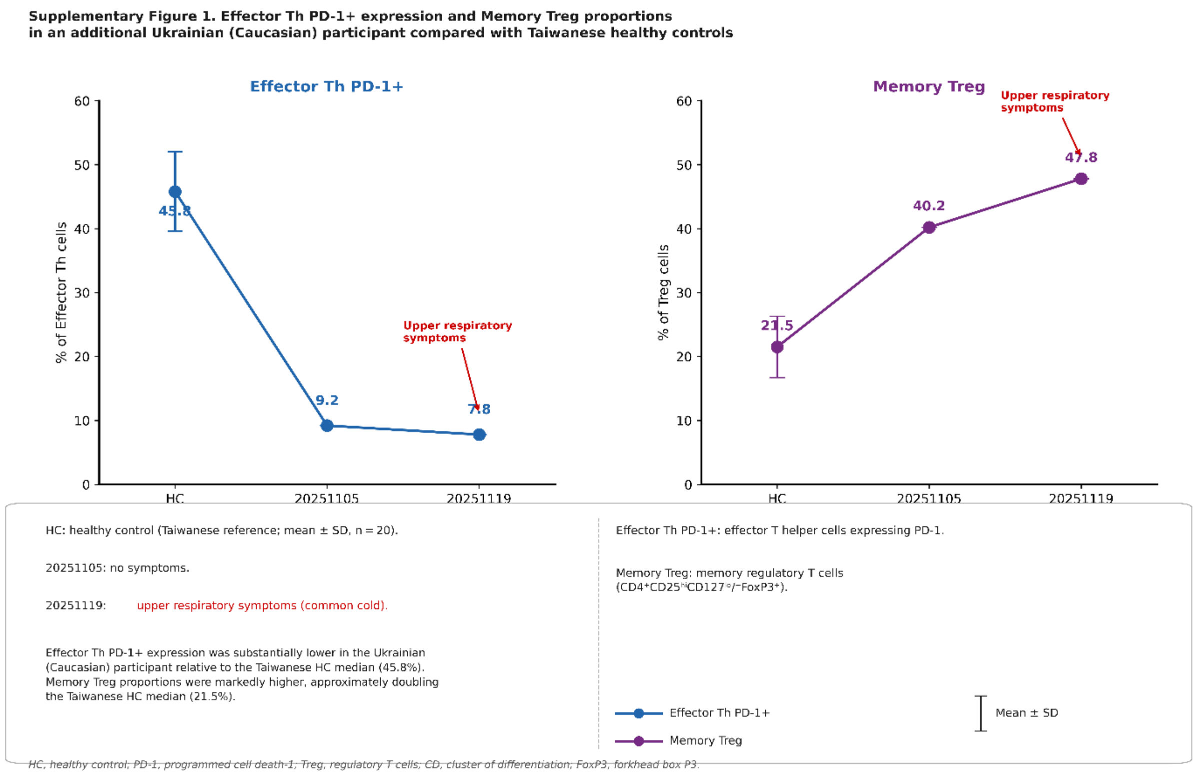

Supplementary Figure 1. Descriptive variation in PD-1^{+} Effector Helper T-cell proportions and Memory Treg proportions in an additional Ukrainian participant.

Serial observations from an additional healthy participant of Ukrainian background are shown relative to the healthy Taiwanese reference cohort (HC, n = 20). PD-1^{+} Effector Helper T-cell proportions and Memory Treg proportions were evaluated during an asymptomatic state (20251105) and a later time point associated with mild upper respiratory symptoms (20251119). Error bars for HC represent mean ± standard deviation (SD). Because this observation was derived from a single individual and was included solely for supplementary descriptive illustration, the findings were not incorporated into formal statistical comparisons, inferential analyses, or cohort-level analyses.

Abbreviations: HC, healthy controls; PD-1, programmed cell death-1; Treg, regulatory T cell; SD, standard deviation.
